# Study of the Oxidation Behavior of Fine-Grained Graphite ET-10 by Combining X-ray μCT with Mercury Porosimetry

**DOI:** 10.3390/nano12244354

**Published:** 2022-12-07

**Authors:** Yumeng Zhao, Yujie Dong, Yangping Zhou, Zhengcao Li, Rui Yan, Zuoyi Zhang

**Affiliations:** 1 Department of Engineering Physics, Tsinghua University, Beijing 100084, China; 2 Institute of Nuclear and New Energy Technology, Tsinghua University, Beijing 100084, China; 3 State Key Laboratory of New Ceramics and Fine Processing, Key Laboratory of Advanced Materials (MOE), School of Materials Science and Engineering, Tsinghua University, Beijing 100084, China

**Keywords:** fine-grained graphite, nuclear graphite ET-10, porosity distribution, gas velocity distribution, ASTM D7542, X-ray μCT, mercury porosimeter, SiC coating, ratio of surface area to volume

## Abstract

By combining X-ray micro-computed tomography with mercury porosimetry, the evolution of the oxygen supply, porous structure, mass loss and oxidized compositions were investigated to characterize the oxidation behavior of fine-grained graphite ET-10, regarding the geometry of the specimen and its oxidation temperature. Here, the porous structure and the gas flows out of and into the porous structure were comprehensively compared for two kinds of specimens—large pure graphite (D = H = 25.4 mm), oxidized at a test facility based on ASTM D7542, and small partially SiC-coated graphite (D ≈ 1 mm and H = 1.95 mm), oxidized in the bottom section of a U-type tube. The fine grains and large geometry resulted in small pores and long flow distances, which exhausted the oxygen in the small stream to the interior of the specimen, making its oxidation deviate from the kinetics-controlled regime. In addition, the well-known three-regime theory was reasonably reinterpreted regarding the oxidation of different compositions, binders and fillers. The kinetics-controlled uniform oxidation mainly oxidizing binders is restricted by their limited contents, while the rate of surface-dominated oxidation increases continuously via the consumption of more fillers. Furthermore, we proposed a new design for the test facility used for the oxidation experiment, wherein a partially shielded millimeter specimen can be oxidized in the long straight bottom section of a U-tube, and this will be discussed further in related future studies.

## 1. Introduction

Carbon or graphite materials have been extensively used in various nuclear fission and fusion systems [1,2,3,4,5], as well as in the fabrication of graphene oxide, which is used in various fields [6]. The service state and structural integrity of nuclear graphite components play a significant role in the safety and operating conditions of the related system [7]. Taking a high-temperature gas-cooled reactor as an example, oxidizing impurities in the coolant helium of the first loop during normal operation [8,9] or large amounts of air entering the reactor core due to severe air ingress accidents [8,10,11,12] will cause oxidative corrosion of the graphite materials, resulting in the degradation of the mechanical strength and structural damage. In this field, experimental work involving a test facility or the construction of a model for oxidation usually helps in studying the oxidation behavior of the related nuclear graphite. By discussing the interactions between the oxygen supply, porous structure and mass loss of compositions under different specimen geometries and oxidation temperatures, a new promising test facility was proposed after explaining why the related oxidation experiments with some fine-grained graphite types deviated from the kinetics-controlled regime. In addition, the three-regime theory [13] was reasonably reinterpreted by stressing the oxidation of different compositions of graphite, binders and fillers.

The American Society for Testing and Materials (ASTM) issued the ASTM D7542 standard [14], which recommends the test conditions that should be used to obtain the kinetic parameters for characterizing nuclear graphite and carbon. The conditions were determined via experiments on coarse-grained or medium-grained graphite, NBG-10, PGXW and H4650 [15]. In this way, the sufficiency of the oxygen supply can be guaranteed and the oxidation is kinetics-controlled [16,17,18]. However, when oxidizing the fine-grained graphite, the situations were found to be different. Under conditions close to those of ASTM D7542, the experiments on graphite IG-110 obtained many different values for the activation energy [19,20,21,22,23]. The stability of the oxidation rate at 650–700 °C decreased quickly with the decrease in the grain sizes of different graphite types within 5–10% mass loss [21,24]. The oxidation rate of graphite ET-10 at 725–750 °C increased quickly with the increase in mass loss, even after the mass loss exceeded 5% [25] (p. 7).

Simultaneously, experiments employing a wide range of oxygen supply conditions indicated that the chemically kinetics-controlled temperature regime of the oxidation of graphite IG-110 should be 540–630 °C [19] (p. 186), which is much lower than the acknowledged range of 650–750 °C [26]. Another study found a complex relationship between the gas flow rate (1–10 L/min) and the oxidation rate of the fine-grained graphite NBG-25 at 700 °C [27] (p. 39). Oxidation experiments on graphite IG-110 and graphite ET-10 with insufficient oxygen supply showed that the oxygen supply, e.g., the gas flow rate and oxygen concentration, is proportional to the oxidation rate [9,28]. The Arrhenius fitting approach was improved by refining both the oxygen supply and the micro surface area of the graphite [29].

At present, the criterion for sufficient oxygen supply is that it be around 10 times higher than that consumed via graphite oxidation [18]. For the NBG-10, PGXW and H4650 graphites with coarse or medium grains (≥300 μm) [15], the pores are large, and the criterion is a suitable requirement when their mass loss reaches a certain value, e.g., 5%. In comparison, the grain size (~20 μm) of fine-grained graphite, e.g., ET-10, IG-110 or NBG-25, is around one-fifteenth or less of that of coarse-grained or medium-grained graphite. This results in the small size of the pores of the specimen, even when the mass loss of the specimen reaches 5% or above [30]. Thus, a minor part of the gas flow can penetrate the interior of the specimen. The 10-times criterion is inapplicable to fine-grained graphite, which is confirmed in our present study [25]. In addition, the oxidation rate is closely related to the porosities of the different graphites at low temperatures, while at high temperatures the oxidation rate is mainly determined by the grain size of the graphite [31].

Simultaneously, the three-regime theory has been widely applied to qualify the oxidation behavior concerning the temperature’s effect on the gas diffusion and oxygen supply in porous graphite [9,13,19,28]. If the oxidation of the graphite was kinetics-controlled, it would start with an onset period (around 0–5% mass loss) and then proceed to a quasi-steady state (around 5–10% mass loss), the behavior of which may be strongly related to the ratio of the binder to filler [26] (p. 6). However, no convincing and detailed explanation is available at present.

The above-mentioned details necessitate a comprehensive investigation into the evolution of the oxygen supply, porous structure and mass loss and oxidized composition regarding the specimen’s geometry and oxidation temperature. The X-ray micro-computed tomography (X-ray μCT) technique [32] has been used to discern high-resolution 3D imaging information and to characterize the interior microstructure of specimens without destruction [33,34,35]. Meanwhile, a CFD (computational fluid dynamics) simulation was carried out to observe the distributions of related parameters, e.g., the temperature and gas velocity, inside or outside of the specimen [19,36]. Micro-hydrodynamics simulations [37,38] using the real porous structure obtained from X-ray μCT images can more effectively reveal the gas velocity distribution and the oxygen supply.

Here, oxidation experiments were carried out by oxidizing different geometries of fine-grained graphite ET-10 in different test facilities. The porous structures of the specimens were obtained by combining X-ray μCT with a mercury porosimeter. The evolution of the gas flow and oxygen supply out of and into the porous structure were examined to explain the oxidation behavior of the specimens with different geometries. The three-regime theory was revisited regarding the oxygen supply and the oxidized compositions of the graphite, fillers and binders. After discussing the existing problems with the test facility used for oxidizing the partially SiC-coated millimeter specimen, we proposed a new design for the oxidation experiment to characterize the oxidation behavior of fine-grained graphite.

## 2. Materials and Methods

### 2.1. Test Specimen

**Figure 1:**
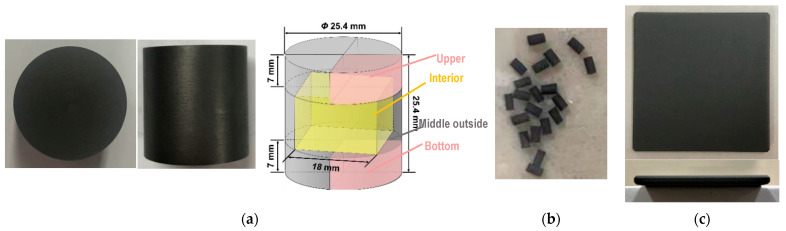
Specimens oxidized in different test facilities: (**a**) large pure graphite specimen; (**b**) small partially SiC-coated graphite specimen; (**c**) fully SiC-coated graphite specimen.

Nuclear graphite ET-10 is high-purity graphite with fine grains (Table 1) [29]. We utilized two kinds of graphite ET-10 specimens—a large pure graphite cylinder and a small partially SiC-coated cylinder (Figure 1a,b and Table 2). The first kind of specimen was provided by IBIDEN Co. Ltd. The second kind of specimen was the product of the fully SiC-coated graphite specimen (Figure 1c) produced by wire cutting, the bottom and upper surfaces of which alone were coated with SiC. After wire cutting, these specimens were rinsed in ethyl alcohol with an ultrasonic cleaner and then dried for 2 h at 120 °C. The fully SiC-coated graphite specimen was also provided by IBIDEN Co. Ltd., Gifu, Japan, whose SiC coating was fabricated via chemical vapor deposition (CVD) [39]. No oxidation occurred when oxidizing the fully SiC-coated specimen at 1200 °C using another test facility [28], which was not considered here. This is in agreement with the fact that the oxidation of SiC will occur until the temperature reaches 1500 °C [40,41,42,43].

Before and after oxidation, the microstructure of the first kind of specimen was observed using a mercury porosimeter (AutoPore IV 9500, Micromeritics). Three parts of the first kind of specimen were measured—the bottom quarter panel, upper quarter panel and interior cuboid (Figure 1a). The air flows up through the bottom surface of the specimen to oxidize the specimen. We adopted X-ray μCT to observe the microstructure of the second kind of specimen. Half of the specimen was used to analyze its porous structure, which was axially divided into five parts. The first part was SiC and the other four parts were graphite.

### 2.2. Oxidation Facility and Experiment Procedure

**Figure 2:**
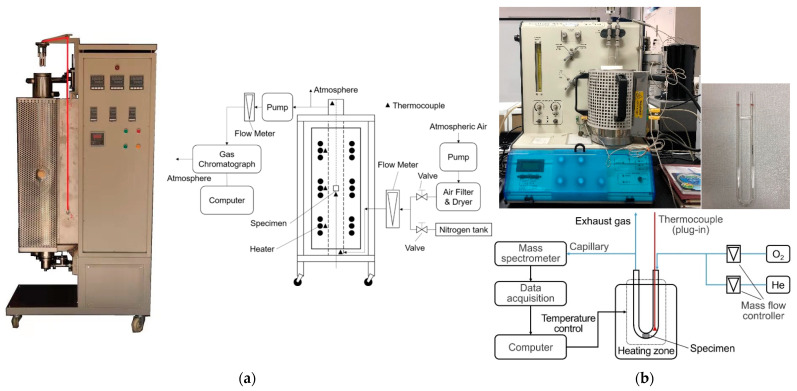
Test facilities used to oxidize different specimens: (**a**) test facility [25] based on recommended conditions of ASTM D7542; (**b**) test facility for oxidizing millimeter specimens in a U-tube.

These two kinds of test specimens were oxidized in two different facilities (Table 2 and Figure 2). The first oxidation facility (Figure 2a) was designed based on the recommended conditions of ASTM D7542 [14]. The description of this test facility can be found in a related study [25] (p. 2).

The second oxidation facility (Figure 2b) was designed for partially SiC-coated millimeter specimens, which are placed at the bottom of a U-tube. The setup mainly comprises a mass spectrometer and a small furnace. The highly purified helium (99.999%) and oxygen (99.995%) are provided by high-pressure tanks. Two mass flow controllers regulate the flow of the mixed helium and oxygen gas into the U-tube. The heating zone is a tank-shaped heater that can quickly heat the specimen at the bottom of the U-tube. The temperature of the specimen detected by one Pt-Rh thermocouple is used to regulate the power of the heater automatically. A mass spectrometer (GSD 320 T1, Thermostar, Velden, Austria) measures the components of the exhaust gas. The masses of the specimen before and after oxidation are obtained from the analytical balance (ML204T/02, Mettler Toledo, Columbus, OH, USA).

These two test facilities have the same test procedure. Firstly, inert gas (nitrogen or helium) is injected into the quartz tube and then the temperature of the specimen is heated to a target value. Then, the oxidizing gas (air or mixed gas of He and O_2_) is driven into the tube to oxidize the specimen. After oxidation, the test specimen is cooled by charging the inert gas (nitrogen or helium) into the tube.

### 2.3. Oxidation Rate and Porosity

The oxidation rate of the first kind of specimen was calculated using the same method as that in the previous study [25]. The oxidation rate of the second kind of specimen was calculated according to Equation (1):

(1) $$OR=\frac{m_{0}-m}{m_{0}t}\times 100\%$$

where $m_{0}$ (mg) = the mass of the specimen before oxidation; m (mg) = the mass of the specimen after oxidation; t (min) = the oxidation time.

The oxidation rate is influenced by the geometry of a specimen, the flow rate of gas and the oxygen concentration of gas. Before comparing the oxidation rates of different specimens oxidized at different facilities, we can correct them by considering the related factors according to Equation (2):

(2) $$OR^{\prime }=\frac{OR}{vC}{\left(\frac{V_{0}}{S_{0}}\right)}^{-\alpha }$$

where $V_{0}$ (mm^3^) = the volume of the specimen before oxidation; $S_{0}$ (mm^2^) = the surface area of the specimen before oxidation; α = the correction factor of the ratio of the surface area to the volume of the specimen before oxidation; v (dm/min) = the flow rate of the gas; and C (mol/L) = the volume concentration of oxygen.

The porosity of a specimen or a part of it can be obtained according to its density obtained using a mercury porosimeter, as in Equation (3):

(3) $$P=1-\frac{\rho }{\rho_{0}}$$

where ρ (mg/mm^3^) = the density of the pure graphite specimen or a part of it; $\rho_{0}$ (mg/mm^3^) = the skeletal density of the graphite, valued at 2.272.

### 2.4. X-ray μCT

**Figure 3:**
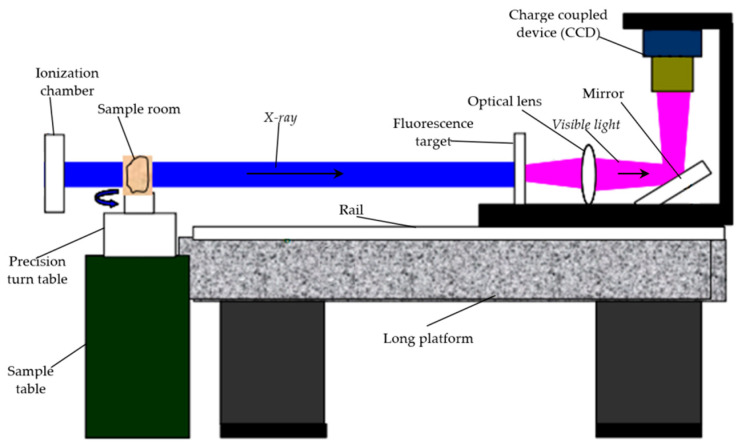
Scheme of the X-ray μCT system at the Shanghai Institute of Applied Physics.

We used the X-ray μCT at Shanghai Institute of Applied Physics (Figure 3). X-rays are generated in an ionization chamber and are transmitted through the specimen in the sample room. Visible light is emitted when a fluorescence target absorbs the X-ray. Then, the visible light goes through an optical lens and a mirror and is captured as a 2D image by a charge coupled device (CCD) camera. When the precision turn table rotates a certain degree or the CCD camera moves a small distance along the rail, another 2D image is taken by the CCD camera. Finally, a set of 2D images is obtained for a specimen. These 2D images are imported, processed and analyzed using the Avizo software (ThermoFisher) to observe the microstructure and the micro-hydromechanics of the specimen.

The values of the related parameters of the X-ray μCT are shown in Table 3. We took pictures of the specimen from the upper surface of the SiC component to the middle of the graphite component. For each specimen, 2048 images were captured and the distance between two neighboring images was 600 nm.

### 2.5. Processing and Analyzing Images

For each specimen, the 2048 2D images were loaded and a 3D image of more than half of the specimen was constructed using the Avizo software. Then, the histogram equalization operation was performed for background correction of the 3D image. After this, we sought to recognize the elements of a 3D gray image as a solid SiC/graphite or pore/air system by setting a threshold (segmentation by interactive thresholding). For the 3D images of different specimens, the thresholds were different.

In addition, because the boundary between the SiC/graphite and air was indistinct and could not be established automatically using the Avizo software, we had to manually outline the boundary using the software. However, it is difficult and very labor-intensive to manually outline all boundaries in numerous 2D images. We axially divided the 3D image into five parts and assumed that all 2D images in each part had the same boundary. The first part was the 3D image of the SiC component, whose height is around 90 μm. The second part was the graphite component close to the SiC, whose height is 165 μm. The other three parts were the graphite components, whose heights equaled 240 μm. The 3D images of the four graphite parts were assembled for further analysis.

Furthermore, after the X-ray–visible light passes through different units, e.g., the sample room, air, fluorescence target, optical lens and mirror, to reach the CCD camera, the light may diverge or concentrate, which finally alters the specimen’s physical size in the CCD camera. In this way, the actual sizes of the pixels in 2D images taken by the CCD camera may be a little different from their theoretical values shown in Table 3.

Here, we propose a mathematical method to determine the actual sizes of the pixels in the 2D images and the values of the related thresholds. Several hypotheses are offered:

- The SiC part and the graphite part of the specimens before oxidation are cylinders, and the differences in their diameters are the same;
- The shape of the SiC part in the specimen is not changed after being oxidized. Its mass loss due to oxidation is determined by the oxidation time;
- The whole specimen can be divided axially into two halves, and they are symmetrical.

The areas of the pixels in the 2D images for different specimens (unoxidized or oxidized at different temperatures) can be obtained using Equation (4):

(4) $$S=\frac{m_{0}}{n_{1}\rho_{1,0}h_{1}+{\left(\sqrt{n_{1}}-4.6\right)}^{2}\rho_{2,0}h_{2}}$$

where $m_{0}$ (mg) = the mass of the unoxidized specimen; $n_{1}$ = the area (the number of pixels) of the middle cross-section of the SiC part in the unoxidized or oxidized specimen; $\rho_{1,0}$ (mg/μm^3^) = the density of the SiC part of the unoxidized specimen; $h_{1}$ (μm) = the height of the SiC part of the unoxidized specimen; $\rho_{2,0}$ (mg/μm^3^) = the density of the graphite part of the unoxidized specimen; and $h_{2}$ (μm) = the height of the graphite part of the unoxidized specimen.

Then, the mass of the SiC part of the unoxidized specimen can be calculated according to Equation (5):

(5) $$m_{1,0}=n_{1}S\rho_{1,0}h_{1}$$

The mass of the SiC part in the oxidized specimen can be calculated according to Equation (6):

(6) $$m_{1}=m_{1,0}\beta$$

where β = the oxidation coefficient of the SiC part of the specimen.

Here, β is obtained as follows:

(7) $$\beta =1-0.75t\;\left(t=4,\;8\right)$$

(8) $$\beta =0.96-0.25t\;\left(t=12,\;16,\;24\right)$$

The average density of the graphite parts in the oxidized specimen can be calculated according to Equation (9):

(9) $$\rho_{2}=\frac{m-m_{1}}{n_{2}Sh_{2}}$$

where $n_{2}$ = the average area (the number of pixels) of the cross-sections of the four graphite parts in the oxidized specimen.

We derived the values of the density and the open porosity of graphite ET-10 by measuring the first kind of specimen with a mercury porosimeter. Then, the expression of the open porosity and density of graphite ET-10 were derived via the polynomial fitting of these values. The expression was applied to calculate the average open porosity of the graphite parts according to the density obtained using Equation (9).

Meanwhile, we varied the threshold for recognizing the elements of a 3D gray image as a graphite solid or pore, and also obtained the average open porosity of the graphite parts with the Avizo software. If the open porosity was equal to that obtained by the above-mentioned expression, the threshold was deemed suitable for the related specimen.

### 2.6. Pore Structure Analysis

After segmentation using the thresholds obtained in Section 2.5, we could analyze the pore structures of the specimens. The radial and axial distributions of the porosity, the open porosity and the closed porosity could in this way be revealed for different specimens.

**Figure 4:**
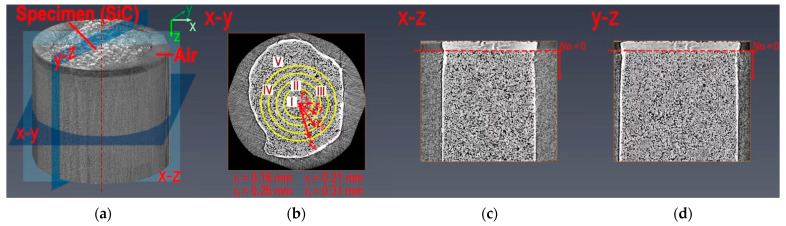
A 3D picture and its different planes of half of the specimen coated by the SiC. (**a**) 3D picture; (**b**) x-y plane; (**c**) x-z plane; (**d**) y-z plane.

A 3D picture of the specimen is shown in Figure 4a, derived after assembling the 2D images captured via X-ray μCT, straightening the specimen and removing the high-luminance corners. When discussing the radial distributions of various porosities of graphite, the specimens are divided into four rings and one cylinder (Figure 4b). When introducing the axial distributions of various porosities of graphite, the junction of the SiC and graphite is set to 0 (Figure 4c,d).

### 2.7. Simulation of Micro-Hydromechanics

The 3D images of the specimens were reprocessed for the simulation of the micro-hydromechanics, where the gas flows through the specimen in the tube.

We employed porous graphite structures in the small partially SiC-coated graphite specimens, including the unoxidized specimen and the specimens oxidized for 4–24 min. In this way, the penetration of the gas flow into the graphite specimens could be observed regarding the porous structures of the different specimens.

**Figure 5:**
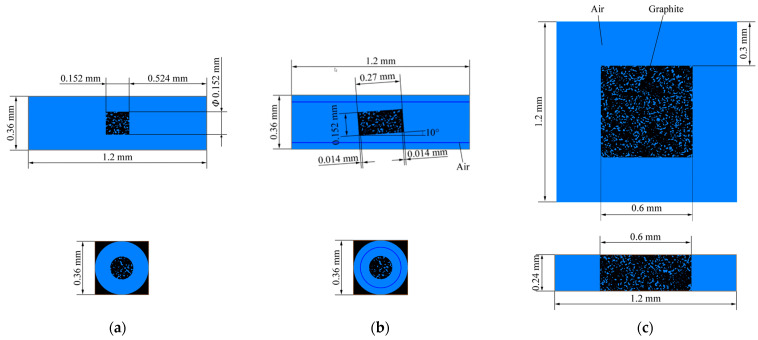
Scaled-down scenarios for the micro-hydromechanics simulation using the real porous structure of graphite ET-10: (**a**) the scaled-down scenario based on the recommended conditions from the ASTM; (**b**) the scaled-down scenario for the partially SiC-coated graphite specimen; (**c**) the scenario for the large pure graphite specimen.

Since a micro-hydromechanics simulation of the real scenario in the test facility was impossible given our available computing ability, three scaled-down scenarios were adopted (Figure 5). These scenarios assumed a porous structure in the interior of the graphite.

Figure 5a simulates the scaled-down scenarios for a pure graphite specimen based on the recommended conditions of ASTM D7542. Figure 5b simulates the scaled-down scenarios of the partially SiC-coated graphite specimen. Figure 5c depicts the scenario for a large pure graphite specimen.

The configurations of the simulation calculation are shown in Table 4. We adopted the Absolute Permeability Experiment Simulation in the Avizo software for the small pure graphite specimen and the small partially SiC-coated graphite specimen (Figure 5a,b). We rotated the unoxidized specimen counterclockwise by 10 degrees to simulate the small angle between the gas flow and the SiC-coated graphite specimen in the bottom section of the U-tube. The fluid viscosity of the gas was set to 4.18 × 10^−5^ Pa·s, which is that of air at 700 °C. The average gas flow rate in front of the specimen was around 18 cm/s, which was close to the values found in the related test facilities when the oxidation temperature was 700 °C. The third scenario used the Absolute Permeability Tensor Calculation for the qualitative analysis.

## 3. Results

### 3.1. Oxidation Rate

**Figure 6:**
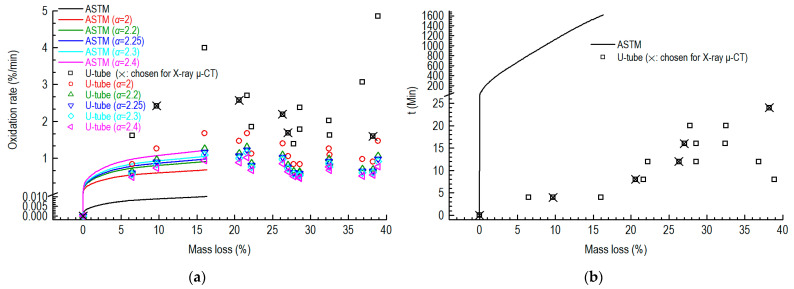
Oxidation rates and times versus mass losses of related specimens oxidized at 700 °C: (**a**) oxidation rates versus mass losses; (**b**) oxidation times versus mass losses (α: correction factor).

We obtained the oxidation rates for the first facility (ASTM D7542, large pure graphite specimens) and the second facility (U-tube, small partially SiC-coated graphite specimens) at 700 °C (Figure 6a).

The oxidation rates of the small specimens were around 1.5–5%/min, which were much faster than those of the large specimens. After the mass loss exceeded 5%, the oxidation rates of the large specimen based on the conditions of ASTM D7542 were around 0.007–0.01%/min. After correcting the rates with Equation (2), they became close, especially when the correction factor (α) was equal to 2.25.

Figure 6b shows the oxidation times versus mass losses of the specimens. More than one day will be required for the first test facility, based on the conditions of ASTM D7542, when the mass loss of the specimen exceeds 15% at 700 °C. The second test facility only requires several minutes.

We chose the six specimens (marked by crosses in Figure 6) for the microstructural observations using X-ray μCT.

### 3.2. Porosities of Pure Specimens Assessed using a Mercury Porosimeter

The large pure graphite specimens were oxidized in the first test facility based on the recommended conditions of ASTM D7542. Three parts of the specimen (Figure 1a)—the bottom, interior and upper parts—were measured using a mercury porosimeter to obtain their densities and other information related to their microstructure. Two groups of specimens were measured at different times, in which the interior part was only measured in the specimens of the second group.

**Figure 7:**
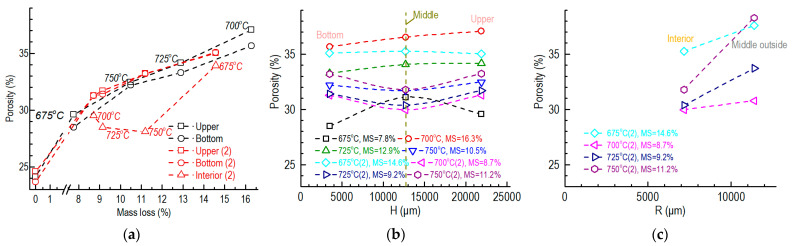

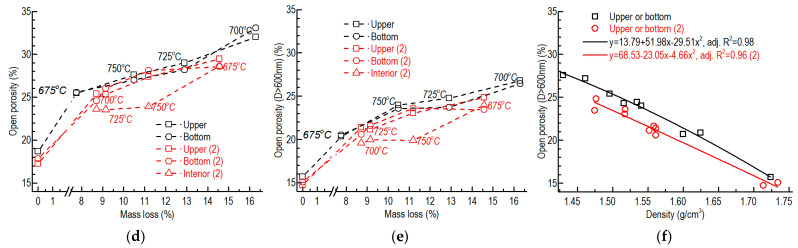
Porosities of pure graphite specimens oxidized based on the conditions of ASTM D7542: (**a**) porosities of different parts versus mass loss of whole specimen; (**b**) axial distribution of the porosity of the specimen; (**c**) radial distribution of the porosity of the middle part of the specimen; (**d**) open porosities of different parts versus mass the loss of the whole specimen; (**e**) open porosities (D > 600 nm) of different parts versus the mass loss of the whole specimen; (**f**) fitting results of the open porosities (D > 600 nm) and densities of different upper and bottom parts.

The porosities of the different parts of the specimen were calculated according to Equation (3), using the densities of the specimens obtained by the mercury porosimeter (Figure 7a–c). The open porosities of the different parts were directly measured using the mercury porosimeter (Figure 7d,e).

According to Figure 7a,b,d, the porosities and the open porosities of the upper and bottom parts were similar for all specimens oxidized at different temperatures. They were mainly determined from the mass loss of the whole specimen (around 5–15%), regardless of the oxidation temperature. When the mass loss of the specimen was close to 5% or 15%, the difference became somewhat larger.

However, the porosities and open porosities of the middle parts were often different from those of the upper and bottom parts at all temperatures (Figure 7a,b,d). When only considering the middle parts, the porosities of the interior were smaller than those of the middle outside parts, and the difference increased with the increases in mass loss and oxidation temperature (Figure 7c).

Since the actual sizes of the pixels in the CCD images taken via X-ray μCT were around 600 nm, we obtained the porosities of the pores whose diameters were larger than 600 nm, as shown in Figure 7e. We derived the expressions of the open porosity (D > 600 nm) and density of graphite ET-10 (Figure 7f) with the method mentioned at the end of Section 2.5.

### 3.3. 3D Pictures of SiC-Coated Graphite Specimen Captured by X-ray μCT

**Figure 8:**
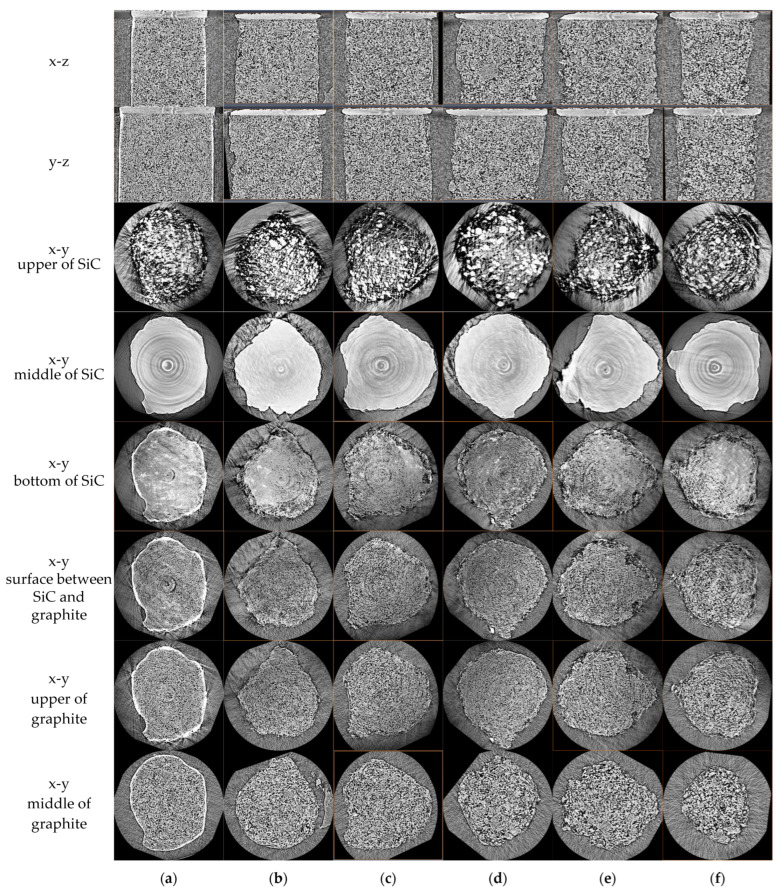
Cross-sections of 3D pictures of partially SiC-coated graphite specimens: (**a**) as-received; (**b**) oxidized for 4 min; (**c**) oxidized for 8 min; (**d**) oxidized for 12 min; (**e**) oxidized for 16 min; (**f**) oxidized for 24 min.

We assessed different sections of the 3D pictures of the SiC-coated graphite specimens (Figure 8). Because the SiC is a high-hardness material that is hard to process via wire cutting, the sections of these specimens were not presented exactly as round faces, even for the unoxidized specimen.

The upper and middle cross-sections of the SiC components corresponded to those obtained via scanning electron microscopy (SEM) observations in another study [39] (p. 4). The upper cross-sections of the SiC components were uneven and the middle cross-sections were uniform, despite the ring artifacts, due to possible problems with the CCD camera.

A transition region (Si_x_C, 0 < x < 1) was present between the SiC component and the graphite component (the fifth to seventh lines of Figure 8). When the oxidation time was short (4–8 min), the oxidation of the transition region was quicker than that of its adjoining graphite region (the first and second lines of Figure 8b,c). The oxidation of this region was slower when the oxidation time reached 12–24 min (the first and second lines of Figure 8d,f).

The above-mentioned observations indicate that the SiC coating has two opposite effects on the oxidation. One is the acceleration of the carbon dust, which is infused into the pores of graphite during the CVD coating process. The other is the shielding effect of the SiC coating, which can prevent the gas from penetrating the pores of the graphite. The first effect is dominant at the beginning of oxidation when the geometries of the graphite component and SiC component are close. The second effect will be dominant when the differences in their geometries increase.

The border region of the unoxidized specimen has lower porosity where the carbon dust is produced during wire cutting and penetrates the pores (Figure 8a). This region will be oxidized first (Figure 8b).

### 3.4. Pore Structure Analysis by Combining X-ray μCT with Mercury Porosimetry

#### 3.4.1. Porosity of Unoxidized Graphite Component Far Away from the SiC Coating

Since the coating process of the SiC can influence the porosity of the graphite, we assessed the graphite part far away from the SiC coating to obtain the porosity of unoxidized pure graphite by processing the 3D picture with the Avizo software. In addition, because the wire cutting process can also influence the porosity of the border of the graphite specimen (Figure 8a), we only used the inner cylinder part of the specimen, whose diameter is 0.7 mm.

**Figure 9:**
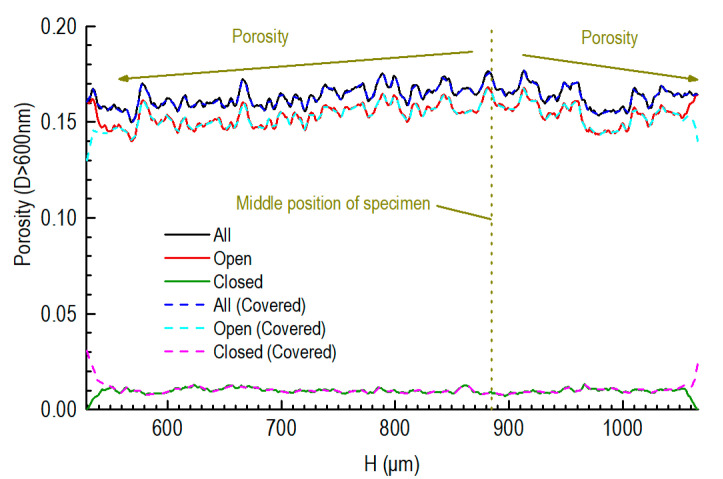
Axial porosity profile of unoxidized graphite component far away from the SiC coating.

When the threshold was set to 34 to recognize the elements of the 3D gray image as solid graphite or pores, the open porosity (D > 600 nm) of the inner cylinder was 15.4%. This value was close to that of the unoxidized graphite specimen obtained by the mercury porosimeter, valued at 15.3%. Figure 9 shows the axial porosity profile of the inner cylinder. The middle position of the specimens was 885 μm away from the interface between the SiC and graphite. From the middle position to the position close to the SiC component, the porosity and the open porosity of the graphite showed a trend of decline. Carbon dust was infused into the pores of the graphite during the CVD coating process.

We also show the axial porosity profile for when the upper and bottom surfaces of the cylinder were covered with SiC, which caused some open pores to close. Thus, the open porosities of graphite closed to the covered surface decrease, while their closed porosities increased.

#### 3.4.2. Porosity of Half of the Partially SiC-Coated Graphite Specimen

**Figure 10:**
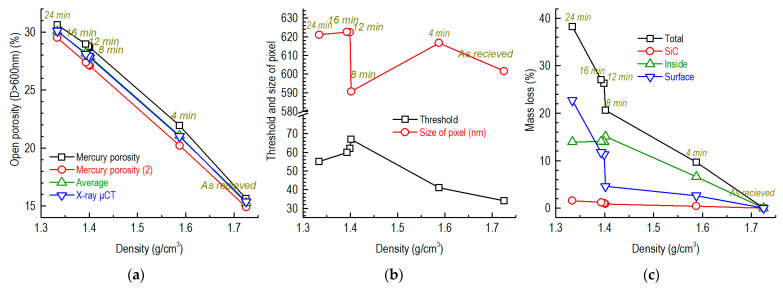
Open porosity, mass loss and related parameters versus density of partially SiC-coated graphite: (**a**) open porosity (D > 600 nm) versus density; (**b**) threshold and size of pixels versus density; (**c**) mass loss versus density.

We obtained the open porosities of half of the oxidized specimens using the Avizo software (Figure 10a). The related threshold mentioned at the end of Section 2.5 was modified until the open porosity (D > 600 nm) was close to that calculated using the fitting expressions (Figure 7f). The thresholds for the different specimens were 34–67 (Figure 10b). The sizes of the pixels in the pictures obtained by X-ray μCT were 591–623 nm, which were below the theoretical value of 740 nm (Table 3). For the specimens oxidized for 4–8 min, the mass loss in the pores comprised the majority of the specimen’s mass loss (Figure 10c). In the specimen oxidized for 12–16 min, the oxidation of the graphite’s surface increased. In the specimen oxidized for 24 min, the oxidation of the specimen’s surface comprised the majority of the specimen’s mass loss.

**Figure 11:**
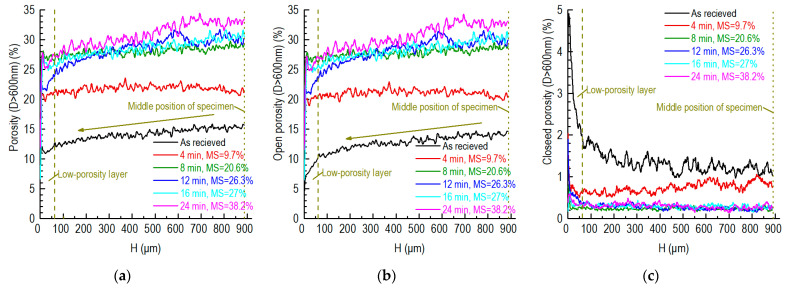
Axial profiles of porosities of partially SiC-coated graphite specimens: (**a**) porosity; (**b**) open porosity; (**c**) closed porosity (MS: mass loss).

The axial profiles of the different porosities are shown in Figure 11 for the graphite component of the partially SiC-coated specimens. The middle position of the specimen was around 885 μm away from the interface between the SiC and graphite.

From the middle position to the position close to the SiC, a trend of decline is shown pertaining to the porosity and the open porosity of the unoxidized graphite, which is the same as that shown in Figure 9. Specifically, a low-porosity layer is present, the thickness of which is around 60 μm. In addition, this low-porosity layer has a highly closed porosity. These features can also be explained by the carbon dust penetrating the pores of the graphite during the CVD coating process.

For the specimens oxidized for 4–8 min, whose mass losses were around 10–20%, the distributions of the porosity and open porosity were even. For the specimens oxidized for 12–24 min (26–38% of mass losses), the porosity and open porosity decreased from the middle position to the position close to the SiC.

**Figure 12:**
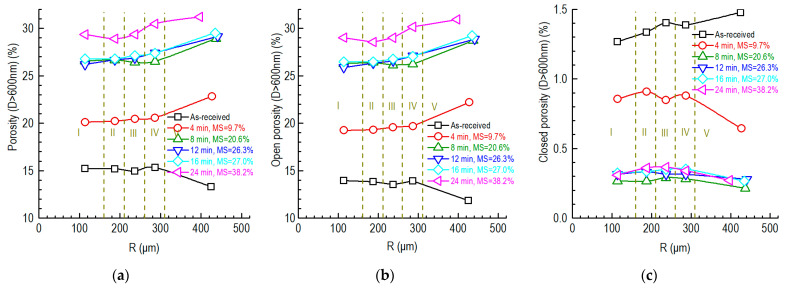
Radial profiles of porosities of partially SiC-coated graphite specimens: (**a**) porosity; (**b**) open porosity; (**c**) closed porosity (MS: mass loss).

Figure 12 shows the radial porosity profiles of the graphite component of the partially SiC-coated specimens. The porosity and open porosity increased with the increase in oxidation time and mass loss. In general, for the specimens oxidized for 4–8 min with around 10–20% mass losses, the porosities of the graphite components were smooth, except for the most outside region (V region in Figure 12).

The outermost region of the unoxidized specimen showed the lowest porosity and open porosity and the highest closed porosity. By comparison, for the oxidized specimen, the outermost region tended to have the highest porosity and open porosity and the lowest closed porosity. In addition, the increases in the porosity and open porosity of the second-most outer region (IV region in Figure 12) were faster than in the inner regions with the increase in oxidation time. The closed porosities were similar when the oxidation times were 8–24 min.

#### 3.4.3. Geometry of Pores

**Figure 13:**
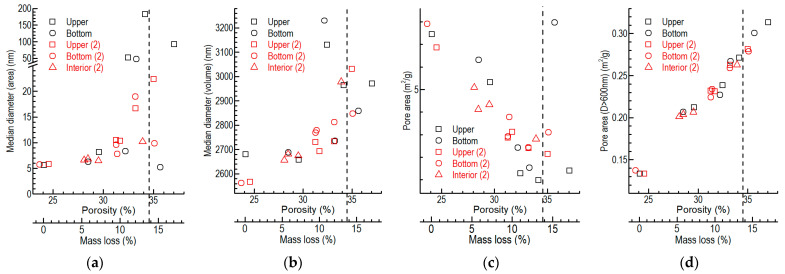
Diameter and pore area versus the porosity and mass loss of the specimen: (**a**) median diameter (area); (**b**) median diameter (volume); (**c**) pore area; (**d**) pore area (D > 600 nm).

The median diameter and the pore area versus the porosity and the mass loss of the different parts of the pure graphite specimen were obtained using the mercury porosimeter (Figure 13). The median diameters of the pores were small; their values were less than 200 nm (regarding the pore area) and 3250 nm (regarding the pore volume).

The median diameter of the pore of graphite ET-10 often increased when the porosity and mass loss increased with the oxidation process. The increase in the median diameters stopped when the porosity reached around 34.5% and the mass loss of the specimen was 13.7%. The pore area often decreased with the increase in pore volume (porosity) until the porosity reached around 34.5%. By comparison, the surface areas of the related large pores (d > 600 nm) tended to increase. The oxidation of graphite mainly enlarged the small pores until the porosity and the mass loss reached high levels of around 34.5% and 13.7%, respectively.

The binder was oxidized more easily than the filler particles due to its higher open porosity and the presence of impurities [44]. In addition, the binder comprised around 15–20% of the mass of the graphite ET-10. The oxidation process mainly oxidized the in-pore binder between the filler particles, enlarging and removing the small pores. When the binders are almost depleted (mass loss>13.7%), new small pores are produced by oxidizing the filler particles.

This can also explain why the porosity (D > 600 nm) of the partially SiC-coated specimen stopped changing when the oxidation times were 8–16 min (Figure 12). When the porosity (D > 600 nm) and open porosity (D > 600 nm) reached 26–28%, corresponding to 34.5% porosity, the in-pore mass loss was around 14% (Figure 10c). After this point, the oxidation proportion of the graphite’s surface increased following the consumption of more filler particles compared to the in-pore binder (Figure 10c).

### 3.5. Simulation Results of Micro-Hydromechanics

#### 3.5.1. Gas Velocity Distributions of Pure Graphite Specimens

**Figure 14:**
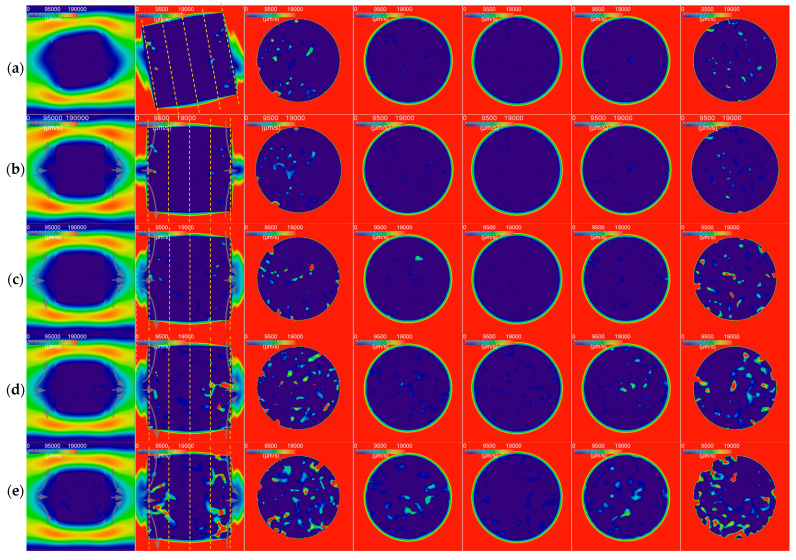
Gas velocity distributions for right cylinder graphite specimens: (**a**) as-received with rotation; (**b**) as-received; (**c**) oxidized for 4 min; (**d**) oxidized for 8 min; (**e**) oxidized for 24 min.

The gas velocity distributions for the pure cylinder graphite specimens are shown in Figure 14. Since the porous structures of the specimens oxidized for 12–16 min were close to that of the specimen oxidized for 8 min, we did not simulate these cases. We also simulated the case by rotating the unoxidized specimen counterclockwise by 10 degrees.

The figures in the first column show the axial sections with a large scale, indicating the gas velocity distributions outside of the specimens. The gas flow on the windward side of the specimen showed three patterns. The two main streams turned up or down into the channel between the specimen and tube. In addition, a part of the gas flow in the channel between the specimen and tube flowed obliquely into the space beyond the leeward side of the specimen.

The gas velocity distributions outside of the specimen were close to those in another study [36] (p. 271). The gas velocity distributions inside the specimen were different since our study adopted the real porous structure of the specimen rather than the fully even porous media.

The figures in the other columns are the axial and radial sections with a small scale, indicating the gas velocity distributions inside the specimens. After penetrating the porous graphite, the small stream also flowed in three ways. The majority of the small stream flowed obliquely into the channel between the specimen and the tube. Similarly, part of it flowed obliquely from the channel between the specimen and the tube into the porous graphite.

In general, the gas flow rates in the unoxidized specimen with a rotation of 10 degrees (Figure 14a) were close to those in the unoxidized specimen without rotation (Figure 14b). The gas flow rates in some pores increased and the gas flow rates in other pores decreased.

The gas velocity inside the pores of the specimen was only around 10% (19,000 (μm/s)/190,000 (μm/s)) of that outside of the specimen. The gas flow rates in the middle regions of the specimens (the fourth to sixth columns of Figure 14) were lower than those in the border regions close to the windward surface or the leeward surface (the third and seventh columns of Figure 14). The difference between the flow rates in the different regions decreased with the increases in oxidation time and porosity. The gas velocity of the flow in the border regions was uniform (the third and seventh columns of Figure 14). By comparison, the velocity of the flow in the middle sub-regions of the middle regions was higher than that of the flow in the border sub-regions (the fourth and sixth columns of Figure 14).

**Figure 15:**
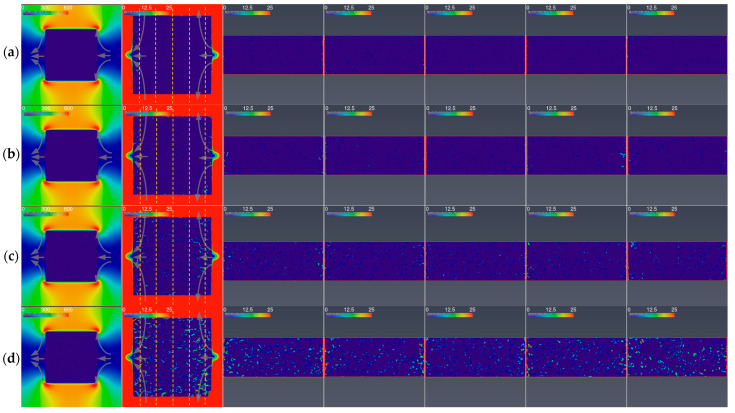
Gas velocity distributions for large cuboid graphite specimens: (**a**) as-received; (**b**) oxidized for 4 min; (**c**) oxidized for 8 min; (**d**) oxidized for 24 min.

The velocity distributions in the large cuboid graphite specimens are shown in Figure 15. The figures in the first column are the sections with a large scale, indicating the velocity distributions outside of the specimens. The gas in front of the windward surface and beyond the leeward surface of the specimen flowed in three ways, similarly to those shown in Figure 14.

The figures in other columns are the sections with a small scale, indicating the velocity distributions inside the specimens. The gas in the border region beyond the windward surface and in front of the leeward surface of the specimen also flowed in three similar ways to those shown in Figure 14.

The gas flow rates in the middle regions of the specimens were lower than those in the border regions close to the windward surface and the leeward surface (the second–seventh columns of Figure 15). The difference between the flow rates in the different regions decreased with the increase in oxidation time and porosity. Differing from those in Figure 14, the gas flow rates in the border sub-regions of the middle and the border regions were all higher than those in the middle sub-regions (the second to seventh columns of Figure 15). The increase in obstructions due to the increase in the size of the specimen made the gas flow in the specimen more border-dominated.

#### 3.5.2. Gas Velocity Distributions for Partially SiC-Coated Graphite Specimens

**Figure 16:**
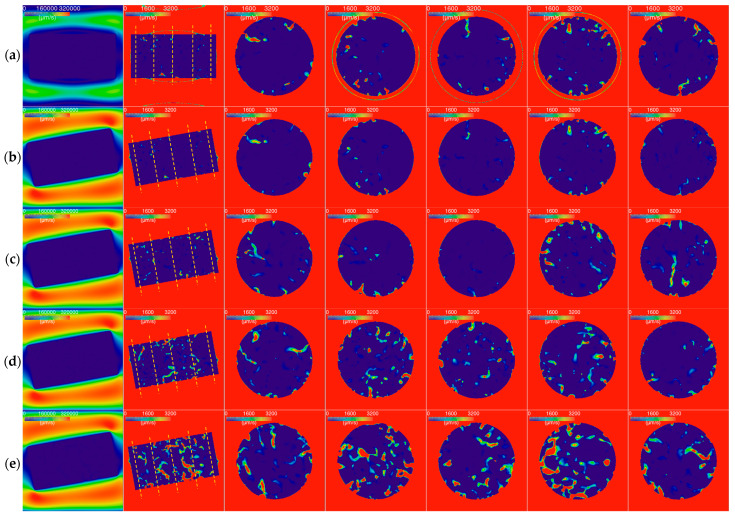
Gas velocity distributions for partially SiC-coated graphite specimens: (**a**) as-received without rotation; (**b**) as-received; (**c**) oxidized for 4 min; (**d**) oxidized for 8 min; (**e**) oxidized for 24 min.

The gas velocity distributions for the partially SiC-coated graphite specimens are shown in Figure 16. We also simulated a case in which the unoxidized specimen was placed horizontally at the center of the tube.

The figures in the first column show the axial sections with a large scale, indicating the gas velocity distributions outside of the specimens. The figures in the other columns are the axial and radial sections with a small scale, indicating the velocity distributions inside the specimens.

In general, the gas flow rate in the unoxidized specimen without rotation (Figure 16a) was higher than that in the unoxidized specimen with a rotation of 10 degrees (Figure 16b). This situation is different from those found with the pure cylinder graphite specimen (Figure 14a,b).

Differing again from those in Figure 14, the gas flow rates in the middle regions of the specimens (the fourth to sixth columns of Figure 16) were close to those in the border regions (the third and seventh columns of Figure 16). The difference between the flow rates in different regions may have been introduced by the randomicity of the porous structure.

The gas flow rates out of the rotated specimens (the first column of Figure 16b–e) were higher than those for the other specimens, namely the unrotated specimen (the first column of Figure 16a) and the pure cylinder graphite specimens (the first column of Figure 14). The gas flow at the tube’s inlet and the inner diameters of the tubes in these cases were set at the same value (Table 4 and Figure 5). In addition, the gas velocity in the pores of the specimen was only 1% (3200 (μm/s)/320,000 (μm/s)) of that outside of the specimen. Thus, the rotated SiC-coated graphite specimens showed a large tangential component of gas flow close to the specimen.

## 4. Discussion

### 4.1. Oxygen Supply, Grain Size and Geometry of Specimen

At present, the criterion for a sufficient oxygen supply is that it should be around 10 times higher than that consumed by the oxidation of graphite [18]. This is a suitable requirement for coarse-grained or medium-grained graphite because a large part of the gas flow facing its windward surface will penetrate the specimen after the onset of the process [26].

However, for fine-grained graphite, e.g., ET-10, the sizes of the pores of the specimens are small, even after the onset of the process when the mass loss is more than 5% and the porosity is more than 28% (Figure 13). The simulation results for the micro-hydromechanics of small specimens (D = H = 0.152 mm) show that the gas velocity in the border regions of the specimen is around 10% of that outside of the specimen (Figure 14). In addition, the majority (more than half) of the gas flow in the border regions close to the windward face of the specimen will bypass the interior region of the specimen (Figure 14). In this way, the gas flowing through the inside of the specimen is around 1% (0.152^2^/0.36^2^ × 0.1/2) of the whole oxygen supply, even without considering the difference in the flow area.

In addition, the geometry of the specimen will also influence the sufficiency of the oxygen supply in two ways. Firstly, for the large specimen, the gas has to flow a long distance to reach the inside, which increases the flow bypassing the middle interior. In addition, the large areas of the windward side and the leeward side of the specimen cause more gas to flow to the border region, increasing the skewness of the radial flow distribution. Thus, even the gas flow in the middle part itself becomes border-dominated, whereby the velocity in the border sub-region is higher than that in the middle sub-region (Figure 15). Secondly, more oxygen will be consumed via oxidation when the gas flow penetrates the pores via a longer distance.

Furthermore, for our micro-hydromechanics simulation shown in Section 3.5, we adopted a uniform porous structure in the middle interior part of the oxidized graphite. The oxidation of graphite usually enlarges the small pores before the porosity and the mass loss reach high levels of around 34.5% and 13.73%, respectively (Figure 13). Hence, the skewness of the flow distribution, oxygen supply and oxidation due to the fine grains and the large geometry will promote each other in an actual oxidation process.

These are the reasons why the porosity of the interior of the large pure graphite specimen is usually less than the porosity of the other parts (Figure 7). In addition, the difference between them increases with the increase in the oxidation temperature, which accelerates the oxygen consumption, especially over the long path to the interior. We can also explain the increased difference between the windward surface and the leeward surface via the increase in oxidation temperature at 675–750 °C [25] (pp. 8 and 9). The oxygen supply at the windward surface is usually sufficient and the oxygen supply at the leeward surface will decrease quickly with the increase in oxidation temperature due to the increased consumption via oxidation.

Here, we can explain the different evolution patterns of the oxygen supply via the extension of pores with the oxidation process in different graphite types based on the recommended conditions of ASTM D7542. At the beginning of the oxidation, the pores are usually small and circular and the gas flow cannot penetrate the interior of the specimen, even with coarse-grained graphite. Then, the small pores are extended and eliminated with the increase in mass loss, resulting in a quick increase in the oxygen supply to the specimen’s interior and the oxidation rate. The oxidation rate of the specimen during the onset process [26], which is mainly determined by the increased oxygen supply, usually increases quickly. For coarse-grained or medium-grained graphite, the pores become large enough when the mass loss reaches a specific level, e.g., above 5%, within the recommended range of oxidation temperatures. The oxygen supply to the interior at this point becomes sufficient, causing the oxidation of the whole specimen to reach a quasi-stable and uniform state, the rate of which is mainly determined by the oxidation temperature. For some fine-grained graphites, e.g., ET-10, IG-110 and NBG-25, the pores are not large enough even when the mass loss reaches 5% or higher at some temperatures within the recommended range, meaning the oxygen supply to the specimen’s interior remains insufficient; that is, the onset process is not completed, meaning the oxidation rate continues to increase with the specimen’s oxidation.

In summary, the oxidation conditions of ASTM D7542 cannot guarantee a sufficient oxygen supply for the oxidation of some fine-grained graphite, e.g., ET-10. The oxidation of graphite ET-10 is not uniform and stable even if the mass loss reaches 5% or higher, which causes it to deviate from the chemically kinetics-controlled regime.

### 4.2. Specialities of Small Partially SiC-Coated Graphite Specimen

For the unoxidized, partially SiC-coated graphite specimen, a declining trend in the porosity and open porosity can be observed from the middle position to the position close to the SiC (Figure 9 and Figure 11). This may be caused by carbon dust penetrating the pores of the graphite during the CVD coating process. In addition, carbon dust can accelerate oxidation in the beginning stage. Of course, further studies are needed to investigate this related topic in greater detail.

The porosity distribution of the small partially SiC-coated graphite specimen is homogeneous, except for in the outermost region (Figure 11 and Figure 12) when the mass losses are around 10–20%. The homogeneous porosity can be explained by the uniform oxidation due to the uniformity of the gas velocity distribution and the sufficient oxygen supply to the small specimen.

The high porosity in the outermost region (Figure 12) can be explained as follows:

- The SiC coating is a high-hardness material and the graphite is a low-hardness material. The uneven side surface and the carbon dust present in it due to the wire cutting (Figure 8) may accelerate the oxidation of the outermost region;
- The angle between the gas flow and the specimen at the bottom section of the U-tube introduces the high-speed tangential component of gas flow close to the specimen (the first column of Figure 16). This also accelerates the oxidation of the outermost region of the specimen.

### 4.3. Conditions for Oxidation Experiment

As mentioned in Section 4.1, the recommended conditions of ASTM D7542 cannot guarantee the oxygen supply required for the oxidation of the fine-grained graphites, e.g., ET-10 and IG-110, even at low temperatures. The oxygen supply and the oxidation of the fine-grained graphite specimen were uneven, deviating from the chemically kinetics-controlled regime, especially for the interior of the specimen.

In addition, oxidation experiments based on ASTM D7542 usually take several hours [25,26]. Experiments on the fine-grained graphite, ET-10, will last more than one day at the lowest temperature. This makes the experiments time-consuming and process-related.

The above-mentioned issues necessitate the improvement of the test facility or the development of a new design. The first choice is to increase the airflow rate. When the oxidation temperature is 700 °C, the gas flow rate of the air in the tube is around 18 cm/s. The increased airflow rate will be more than 1 m/s, which brings lots of problems related to structure and heating. Another choice is to adopt a partially shielded millimeter specimen instead of the large specimen (D = H = 25.4 mm). A previous study also adopted a similar shielding design, but its specimen was still large (D = 21 mm) [19] (p. 184). Our design using partially shielded millimeter-sized specimens has the following advantages for characterizing the oxidation behavior of graphite:

- As shown in Figure 16, the gas flow in the specimen is uniform when its two round faces are covered by SiC;
- The short distance from the surface to the inside of the small specimen means the oxygen in the gas can penetrate the specimen’s interior before being exhausted via oxidation;
- The oxidation rate is quick. It only took several minutes to reach around 10% mass loss at 700 °C. We can repeat the oxidation experiments easily;
- The millimeter specimen facilitates the measurements employed for observing the microstructure of the specimen, e.g., X-ray μCT, SEM;
- The costs for constructing and operating the small test facility are lower than those for a large test facility.

When the gas flow is parallel to the symmetrical axis of the specimen, the gas can more easily penetrate the pores of the specimen (Figure 16a,b). In addition, when the gas flow has an angle of 10 degrees to the symmetrical axis of the specimen, this will result in a high-speed tangential flow close to the specimen (the first column of Figure 16). The high-speed tangential flow increases the oxidation of the surface and the outermost region of the specimen (Figure 10c and Figure 12). Thus, the bottom of the U-tube should include a long straight section to make the gas flow parallel to the symmetrical axis of the specimen.

### 4.4. What Was Oxidized and Its Relevance to the Three-Regime Theory

Nuclear graphite is usually composed of fillers and binders. Studies have noted that the binder can be oxidized more readily due to the higher porosity and impurities [44], which could explain the differences in the oxidation behaviors of the different graphites fabricated via various processes [25]. The binder will be more oxidized by the chemically kinetics-controlled oxidation and more filler will be oxidized with the increase in temperature (see [25] (pp. 8 and 9) and [45] (p. 155)).

At related low temperatures, e.g., 675–750 °C, the oxidation of pure graphite ET-10 mainly increases, and this eliminates the small pores until the porosity and the mass loss reach high levels of 34.5% and 13.7%, respectively. (Figure 13). The overall and SEM pictures of the oxidized specimens in our previous study also showed a similar situation [25] (pp. 6, 8 and 9). The mass losses of the in-pore oxidation of graphite were usually less than 15% (SiC-coated graphite, Figure 10c; pure graphite, Figure 13), which comprises the majority of the oxidation. When the mass loss of graphite ET-10 was higher than around 15% at 700 °C, the increases in the mass loss were mainly caused by surface oxidation (Figure 10c).

The binder comprises 15–20% of the mass of the graphite ET-10, which is oxidized more easily than the filler. We can infer that the oxidation of graphite ET-10 at 675–750 °C in the two test facilities mainly consumed the binder of the graphite when the mass loss was less than 15%. When the mass loss of graphite ET-10 is higher than around 15% or the oxidation temperature is higher than 750 °C, the oxidation will be more surface-dominated, whereby the oxidation of the filler grain will increase.

**Figure 17:**
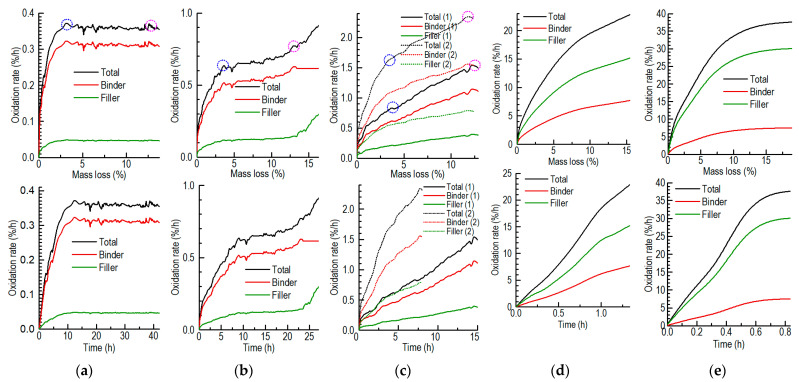
Oxidation rates of the whole specimen and binders and fillers of graphite ET-10 at 675–900 °C based on ASTM D7542: (**a**) 675 °C (chemically kinetics-controlled, regime I); (**b**) 700 °C (kinetics and in-pore diffusion mixed); (**c**) 725 °C (1) and 750 °C (2) (in-pore diffusion-controlled, regime II); (**d**) 850 °C (in-pore diffusion and boundary layer mixed); (**e**) 900 °C (boundary layer-controlled, regime III).

At present, the three-regime theory is usually applied to approximate the temperature’s effect on the oxidation behavior regarding the balance between the gas diffusion and oxygen consumption of porous materials. Here, we can reveal more convincingly the oxidation behavior regarding the changes in oxidized compositions and the increase in mass loss in an actual oxidation process, including the onset process [26]. We revisited the three-regime theory by using the real evolution of the oxidation rate of the graphite ET-10 oxidized at 675–900 °C under the conditions recommended by ASTM-D7542 (Figure 17). A similar situation can also be found in a previous study [21] (p. 3359). By supposing different suitable ratios of the oxidation rate of the binder to that of the filler at different temperatures, the oxidation rates of the binder and filler were also obtained.

The oxidation of graphite at low temperatures is chemically kinetics-controlled and mainly consumes the binder (Figure 17a). When the mass loss is low, e.g., less than 5% (onset stage), the oxidation rate increases quickly with the increase in oxygen supply penetrating the graphite by enlarging the small pores. Because the binder usually constitutes around 15–20% of the mass of the graphite, the decreased binder, enlarged pores and increased oxygen supply reach a balance when the mass loss is around 5–10%. Then, the oxidation rate becomes stable. After the mass loss of graphite increases and the binder is exhausted, the oxidation of the filler will increase by creating new pores.

When the temperature is high (in-pore diffusion-controlled regime), the filler grain can be oxidized more easily (Figure 17c). The surface oxidation process simultaneously consuming the filler and binder is comparable to the in-pore oxidation that mainly consumes the binder. The consumption of the binder only comprises a part of the mass loss that extends the onset stage. Namely, when the mass loss of the specimen is higher than 5% or even 10%, the consumption of the binder is low so the enlargement of the small pores continues via the oxidization of the in-pore binder. Thus, the oxidation rate increases continuously, even if the mass loss is higher than 10%.

When the oxidation mainly oxidizes the binder (Figure 17a–c), the increase in mass loss of the binder even at a low level may influence the oxidation rate because the binder comprises a small proportion of graphite, e.g., 15–20% of the mass of graphite ET-10. When the mass loss is around 3%, trend changes in the oxidation rate occur (blue cycles in Figure 17a–c). In addition, when the mass loss is around 13%, other trend changes in the oxidation rate also occur (purple cycles in Figure 17a–c). This corresponds with the cessation of the increase in the in-pore oxidation at around 14% of the mass loss (Figure 10c) and the trend changes in the geometry of the pores after 13.7% of the mass loss (Figure 13a–c). This is also the reason why the oxidation rate is closely related to the porosities of different graphites at low temperatures [31] (p. 5), because the binder is much more porous than the filler grains.

When the temperature is very high (when the in-pore diffusion and boundary are layer mixed, as in Figure 17d, or the boundary layer is controlled, as in Figure 17e), oxidation mainly occurs on the surface, which simultaneously oxidizes the filler and binder. The oxidation rate of the filler is usually higher than that of the binder due to the high proportion of the filler. The onset process extends the porous structure and simultaneously eliminates the surface region of graphite. When the oxidation rate reaches the limitation of the diffusion rate of the reactants and products near the boundary layers of the filler and binder, it may become nearly stable (Figure 17c). In addition, the reason why the oxidation rate is inversely proportional to the size of the filler grain [31] can be explained by the small surface-to-volume ratios of large objects. This also agrees with our correcting results for the oxidation rates for the different specimens (Equation (2) and Figure 6) if the specimen is considered as a whole object.

### 4.5. Future Studies

Future studies related to this study could focus on two aspects. The first idea is to design a test facility wherein a series of experiments can be carried out efficiently and economically to oxidize fine-grained graphite in the chemically kinetics-controlled mode. The present design used to oxidize the millimeter specimen has two limitations, resulting in the unevenness of the oxidation, especially for the outermost region (Figure 12):

- To process the high-hardness SiC, wire cutting was adopted. However, the side surface of the obtained SiC-coated specimen was very uneven, and a lot of carbon dust penetrated the uneven side’s surface;
- The arc flow path in the bottom section of the U-tube resulted in the high-speed tangential component of the gas flow close to the specimen.

The following efforts may help to overcome these limitations:

- A detachable structure using SiC or other non-corrosive materials will be adopted to cover the two round faces of the small pure cylinder graphite. Pure cylinder graphite can be produced easily with low contents of carbon dust in the even side surface;
- The U-tube will have a long straight bottom section and the specimen will be placed in the middle of the center of the bottom section.

Our present study only considered the fine-grained graphite ET-10 produced using coal tar pitch coke with a non-impregnation baking process. Systematic experiments will be carried out to establish better experimental conditions for the new design in detail, the configurations of which can be roughly predicted as follows:

- Graphite: ET-10, IG-110, etc.;
- Airflow at inlet: 0.005–0.06 L/min;
- Geometry of the graphite specimen: D = 0.5–1.5 mm, H = 1–2 mm;
- Inner diameter of the quartz tube: 3–5 mm;
- Oxidation temperature range: 500–800 °C;
- Observations of the microstructure and impurities: X-ray μCT, mercury porosimetry, SEM, EDX (energy-dispersive X-ray spectroscopy) [6], etc.

The second aspect regards the mechanism analysis, which aims to establish qualitative and quantitative models for the oxidation of the porous graphite. As mentioned in Section 4.4, the chemically kinetics-controlled oxidation of graphite mainly consumes the in-pore binder, with low temperatures and mass losses. With the increase in temperature or mass loss, the oxidation of the filler grain will increase. The oxidation of graphite can be decoupled by dividing it into the oxidation of the binder and the filler grains, whereby some studies in other fields may be referred to, as follows:

- The related discussion of the oxidation mechanism for synthesizing graphite oxide [46] can provide a clue for establishing the model for chemically kinetics-controlled oxidation. Some related studies had been successfully carried out [13]. In addition, the related oxidation processes and oxidizing agents may help to selectively oxidize the binder or the filler for contrast experiments.
- The oxidation mechanism of the filler grain may be relevant to the related kinetics study of the oxidation of highly oriented pyrolytic graphite (HOPG) [47,48]. The properties of HOPG are similar to those of the graphite grain. The beginning stage of the oxidation with a low gas flow rate (0.3 L/min) at 600 °C [49] (p. 86) is similar to that of the HOPG [47] (p. 9946).
- The oxidation of nuclear graphite will be varied with the evolution of the oxidized compositions and the oxygen supply due to the interactions among the intrinsic characteristics and exterior conditions, e.g., the ratios of the different compositions, geometry, microstructure, gas flow rate, temperature, mass loss, etc. The two above-mentioned mechanisms should be integrated to establish a comprehensive model for the oxidation of nuclear graphite.

## 5. Conclusions

By combining X-ray μCT with a mercury porosimeter, the oxygen supply in the porous structure can be compared comprehensively between large pure graphite and small partially SiC-coated graphite specimens. The long flow path will exhaust the oxygen in the narrow stream penetrating the interior of the fine-grained graphite. This can explain why the oxidation of the fine-grained graphite ET-10 may deviate from the kinetics-controlled regime, even under the recommended conditions of ASTM D7542. By comparison, the short flow path and the uniform gas flow maintained a sufficient oxygen supply to the interior of the partially SiC-coated millimeter specimen. In addition, the three-regime theory was revisited to explain the oxidation process by stressing the oxidized compositions of the graphite, binders and fillers. The kinetics-controlled uniform oxidation mainly oxidizes limited binders, whose rate becomes stable with low mass losses, e.g., 5%, while surface-dominated oxidation consuming more fillers extends the onset stage.

The wire cutting process of the SiC-coated graphite and the arc shape of the bottom section of the U-tube introduced additional skewness into the oxidation in the radial direction. In addition, our present study only considered the graphite ET-10 produced using coal tar pitch coke with a non-impregnation baking process. Thus, we propose a new, promising design for the oxidation experiment to characterize the oxidation behaviors of different fine-grained graphites, whereby a millimeter-sized cylindrical graphite sample in a detachable shield structure will be oxidized in the long straight bottom section of a U-tube.

Future studies will include systematic experiments to refine the new design by varying the graphite material, specimen geometry, airflow rate, oxidation temperature, and other factors. Mechanism analyses aiming to establish qualitative or quantitative models may also be carried out by discussing the evolution of the oxidized compositions and the oxygen supply due to the interactions among the various intrinsic characteristics and exterior conditions.

## Figures and Tables

**Table 1:** The main impurities of graphite ET-10 [25].

Impurity	B	Si	Ca	Fe	Al	K	V	Mg
Value (ppm)	<0.1	<1.0	^–^	<1.2	<0.1	^–^	^–^	<0.1

^–^ Not detected.

**Table 2:** The configurations of different graphite ET-10 specimens and related test facilities.

No	Geometry (mm)	Temperature (°C)	Time (min)	Flow Rate (L/min)	Oxygen Concentration (%)	Inner Diameter of Tube (mm)	Thickness of SiC (μm)	Density (mg/mm^3^)	
								Graphite	SiC
1	D = H = 25.4	675–900	480–2520	10	21% (air)	65	\	1.725	\
2	D ≈ 1, H = 1.95	700	4–24	0.04	10% (He and O_2_)	4	90	1.725	2.9

**Table 3:** Configuration of the X-ray μCT system at the Shanghai Institute of Applied Physics.

Item Name	CCD Camera			Number of Images	Distance of Two Images (nm)
	Size of Pixel (nm^2^)	Pixel	Magnification		
Value	740 × 740	2048 × 2048	10	2048	600

**Table 4:** The configurations of the micro-hydromechanics simulation.

Item Name	Absolute Permeability Experiment Simulation				Absolute Permeability Tensor Calculation
	Pressure at Outlet (Pa)	Flow Rate at Inlet (μm^3^/s)	Fluid Viscosity (Pa·s)	Convergence Criterion	Iterations
Value	10^5^	1.9 × 10^10^	4.18 × 10^−5^	1 × 10^−4^	5000

## Data Availability

The datasets generated during the study are at https://github.com/zhouyp97/ET-10-X-ray-CT-mercury-porosimetry.

## References

[1] Irvine J.M. (2011). Nuclear Power: A Very Short Introduction.

[2] U.S. DOE Nuclear Energy Research Advisory Committee, Generation IV International Forum (2002). A Technology Roadmap for Generation IV Nuclear Energy Systems.

[3] Bowden-Reid R., Khachan J. (2021). An inertial electrostatic confinement fusion system based on graphite. Phys. Plasmas.

[4] Tanabe T. (2019). Revisiting carbon materials as plasma-facing material of a fusion reactor. Plasma Phys. Rep..

[5] Goriaev A., Wauters T., Brakel R., Brezinsek S., Dinklage A., Fellinger J., Grote H., Moseev D., Sereda S., Volzke O. (2020). Wall conditioning at the Wendelstein 7-X stellarator operating with a graphite divertor. Phys. Scr..

[6] Alanazi H., Alharbi Y.R., Abadel A.A., Elalaoui O. (2022). Effect of edge oxidized graphene oxide on micro and macro mechanical properties and microstructure of cement paste. Int. J. Mater. Res..

[7] Zhou X., Yang Y., Song J., Lu Z., Zhang J., Liu B., Tang Y. (2018). Carbon materials in a high temperature gas-cooled reactor pebble-bed module. New Carbon Mater..

[8] Yu X., Yu S. (2009). The gasification of graphite matrix in pebble bed reactors. Proceedings of the Volume 2: Structural Integrity, Safety and Security, Advanced Applications of Nuclear Technology, Balance of Plant for Nuclear Applications.

[9] Sun X., Dong Y., Zhou Y., Li Z., Shi L., Sun Y., Zhang Z. (2017). Effects of reaction temperature and inlet oxidizing gas flow rate on IG-110 graphite oxidation used in HTR-PM. J. Nucl. Sci. Technol..

[10] Moormann R., Hinssen H.-K., Kühn K. (2004). Oxidation behaviour of an HTR fuel element matrix graphite in oxygen compared to a standard nuclear graphite. Nucl. Eng. Des..

[11] Chen Z., Chen X., Zheng Y., Sun J., Chen F., Shi L., Li F., Dong Y., Zhang Z. (2017). Air ingress analysis of chimney effect in the 200 MWe pebble-bed modular high temperature gas-cooled reactor. Ann. Nucl. Energy.

[12] Yadav S.K., Joshi M., Sharma Y., Shukla P., Kaushik A., Sapra B.K., Singh R.S. (2019). Physico-chemical characteristics of graphite aerosols generated during postulated air ingress accident. Ann. Nucl. Energy.

[13] El-Genk M.S., Tournier J.M.P. (2011). Development and validation of a model for the chemical kinetics of graphite oxidation. J. Nucl. Mater..

[14] (2015). Standard Test Method for Air Oxidation of Carbon and Graphite in The Kinetic Regime.

[15] Contescu C.I. (2009). Inter-laboratory study to establish precision statements for astm d7542-09, standard test method for air oxidation of manufactured carbon and graphite in kinetic regime. Tech. Rep..

[16] Walker P.L., Rusinko F., Austin L.G. (1959). Gas reactions of carbon. Advances in Catalysis.

[17] Kane J.J., Contescu C.I., Smith R.E., Strydom G., Windes W.E. (2017). Understanding the reaction of nuclear graphite with molecular oxygen: Kinetics, transport, and structural evolution. J. Nucl. Mater..

[18] Contescu C.I., Azad S., Miller D., Lance M.J., Baker F.S., Burchell T.D. (2008). Practical aspects for characterizing air oxidation of graphite. J. Nucl. Mater..

[19] Kim E.S., NO H.C. (2006). Experimental study on the oxidation of nuclear graphite and development of an oxidation model. J. Nucl. Mater..

[20] Lee J.J., Ghosh T.K., Loyalka S.K. (2014). Oxidation rate of nuclear-grade graphite IG-110 in the kinetic regime for vhtr air ingress accident scenarios. J. Nucl. Mater..

[21] Contescu C.I., Guldan T., Wang P., Burchell T.D. (2012). The effect of microstructure on air oxidation resistance of nuclear graphite. Carbon.

[22] Wang P., Yu S.Y. (2012). Effects of gas flow rate and temperature on the oxidation rate of ig-110 nuclear graphite. J. Tsinghua Univ. Sci. Tech..

[23] Wang P. (2013). The Oxidation Performance of IG-110 Nuclear Graphite Used in HTGRs. Ph.D. Thesis.

[24] Chi S.H. (2014). Effects of specimen size on the flexural strength and Weibull modulus of nuclear graphite IG-110, NGB-18, and PCEA. J. Nucl. Mater..

[25] Zhao Y., Dong Y., Zhou Y., Li Z., Zhang Z. (2021). Oxidation experiments and kinetics analysis of nuclear graphite ET-10 by gas analysis and microstructure observation. Energies.

[26] Smith R.E., Kane J.J., Windes W.E. (2021). Determining the acute oxidation behavior of several nuclear graphite grades. J. Nucl. Mater..

[27] Chi S.H., Kim G.C. (2017). Effects of air flow rate on the oxidation of NBG-18 and NBG-25 nuclear graphite. J. Nucl. Mater..

[28] Yan R., Dong Y., Zhou Y., Sun X., Li Z. (2017). Investigation of oxidation behaviors of nuclear graphite being developed and IG-110 based on gas analysis. J. Nucl. Sci. Technol..

[29] Zhou Y., Dong Y., Yin H., Li Z., Yan R., Li D., Gu Z., Sun X., Shi L., Zhang Z. (2018). Characterizing thermal-oxidation behaviors of nuclear graphite by combining O2 supply and micro surface area of graphite. Sci. Rep..

[30] Chi S.H., Kim G.C. (2008). Comparison of the oxidation rate and degree of graphitization of selected IG and NBG nuclear graphite grades. J. Nucl. Mater..

[31] Lo I.H., Yeh T.K., Patterson E.A., Tzelep A. (2020). Comparison of oxidation behavior of nuclear graphite grades at very high temperatures. J. Nucl. Mater..

[32] O’Sullivan J.D.B., Behnsen J., Starborg T., MacDonald A.S., Phythian-Adams A.T., Else K.J., Cruickshank S.M., Withers P.J. (2018). X-ray micro-computed tomography (μCT): An emerging opportunity in parasite imaging. Parasitology.

[33] Sumita J., Shibata T., Fujita I., Kunimoto E., Yamaji M., Eto M., Konishi T., Sawa K. (2014). Development of evaluation method with X-Ray tomography for material property of IG-430 graphite for VHTR/HTGR. Nucl. Eng. Des..

[34] Liu Q., Xu D., Tan C. (2019). Evolution of pore structure and fractal characteristics of graphite lining in rare earth electrolytic cell during high temperature oxidation. Chem. Ind. Eng. Prog..

[35] Cho Y.J., Garcia D., Yu H.Z., Deng Z., Li L., Lu K. (2021). Oxidation behaviors of matrix-grade graphite during water vapor ingress accidents for high temperature gas-cooled reactors. Carbon.

[36] Lu W., Li X., Wu X., Sun L., Li Z. (2020). Investigation on the oxidation behavior and multi-step reaction mechanism of nuclear graphite SNG742. J. Nucl. Sci. Technol..

[37] Hannon L., Lie G.C., Clementi E. (1988). Micro-hydrodynamics. J. Stat. Phys..

[38] Wolf F.G., Santos L.O.E., Philippi P.C. (2008). Micro-hydrodynamics of immiscible displacement inside two-dimensional porous media. Microfluid Nanofluidics.

[39] Hu Z., Zheng D., Tu R., Yang M., Li Q., Han M., Zhang S., Zhang L., Goto T. (2019). Structural control of highly-oriented polycrystal 3C-SiC bulks via halide CVD. Materials.

[40] Zhu Q., Qiu X., Ma C. (1999). Oxidation resistant SiC coating for graphite materials. Carbon.

[41] Tang C., Gong J. (1995). Improvement in oxidation resistance of the nuclear graphite by reaction-coated SiC coating. J. Nucl. Mater..

[42] He Z., Lian P., Song Y., Liu Z., Song J., Zhang J., Ren X., Feng J., Yan X., Guo Q. (2018). Protecting nuclear graphite from liquid fluoride salt and oxidation by SiC coating derived from polycarbosilane. J. Eur. Ceram. Soc..

[43] Yang H., Zhao H., Wang T., Liu X., Zhang K., Li Z., Gao Y., Liu B. (2021). A multi-layered SiC coating to protect graphite spheres from high temperature oxidation in static air. Corros. Sci..

[44] Kim E.S., No H.C., Kim B.J., Oh C.H. (2008). Estimation of graphite density and mechanical strength variation of VHTR during air-ingress accident. Nucl. Eng. Des..

[45] Huang W.H., Tsai S.C., Yang C.W., Kai J.J. (2014). The relationship between microstructure and oxidation effects of selected IG- and NBG-grade nuclear graphites. J. Nucl. Mater..

[46] Li C., Chen X., Shen L., Bao N. (2020). Revisiting the oxidation of graphite: Reaction mechanism, chemical stability, and structure self-regulation. ACS Omega.

[47] Hahn J.R., Kang H. (1999). Mechanistic study of defect-induced oxidation of graphite. J. Phys. Chem. B.

[48] Hahn J.R. (2005). Kinetic study of graphite oxidation along two lattice directions. Carbon.

[49] Choi W.K., Kim B.J., Kim E.S., Chi S.H., Park S.J. (2011). Oxidation behavior of IG and NBG nuclear graphites. Nucl. Eng. Des..

